# Prognostic Factors and Clinical Outcomes of Spontaneous Intracerebral Hemorrhage: Analysis of 601 Consecutive Patients from a Single Center (2017–2023)

**DOI:** 10.3390/neurosci6030077

**Published:** 2025-08-12

**Authors:** Cosmin Cindea, Vicentiu Saceleanu, Victor Tudor, Patrick Canning, Ovidiu Petrascu, Tamas Kerekes, Alexandru Breazu, Iulian Roman-Filip, Corina Roman-Filip, Romeo Mihaila

**Affiliations:** 1 Faculty of Medicine, Lucian Blaga University of Sibiu, 550024 Sibiu, Romania; vicentiu.saceleanu@ulbsibiu.ro (V.S.); tudorvictor2012@yahoo.com (V.T.); ovidiu.petrascu@ulbsibiu.ro (O.P.); breazu.alexandru@yahoo.com (A.B.); corina.roman@ulbsibiu.ro (C.R.-F.); 2 County Clinical Emergency Hospital of Sibiu, 550245 Sibiu, Romania; 3 Limerick University Hospital, V94 F858 Limerick, Ireland; patrickcanning9@gmail.com; 4 County Clinical Emergency Hospital of Brasov, 500097 Brasov, Romania; kerekesj.tamas@yahoo.com; 5 County Clinical Emergency Hospital of Targu Mures, 540136 Targu Mures, Romania; roman-filip.iulian.22@stud.umfst.ro

**Keywords:** intracerebral hemorrhage, hematoma volume, intraventricular hemorrhage, neurosurgery, mortality, neurosurgical treatment, risk factors

## Abstract

Background: Spontaneous intracerebral hemorrhage (ICH) has the highest case fatality of all stroke types, yet recent epidemiological and outcome data from Central and Eastern Europe remain limited. Methods: We retrospectively analyzed prospectively collected data for 601 consecutive adults with primary ICH admitted to Sibiu County Clinical Emergency Hospital, Romania (2017–2023). Demographics, Glasgow Coma Scale (GCS), CT-derived hematoma volume (ABC/2), anatomical site, intraventricular extension (IVH), treatment, comorbidities, and in-hospital death were reported with exact counts and percentages; no imputation was performed. Results: Mean age was 68.4 ± 12.9 years, and 59.7% were male. Mean hematoma volume was 30.4 mL, and 23.0% exceeded 30 mL. IVH occurred in 40.1% and doubled mortality (50.6% vs. 16.7%). Overall case fatality was 29.6% and climbed to 74.5% for brain-stem bleeds. Men, although younger than women (66.0 vs. 71.9 years), died more often (35.4% vs. 21.1%; risk ratio 1.67, 95% CI 1.26–2.21). Systemic hazards amplified death risk: Oral anticoagulation, 44.2%; chronic alcohol misuse, 51.4%; thrombocytopenia, 41.0%; chronic kidney disease, 42.3%. Conservative management (74.9%) yielded 27.8% mortality overall and ≤15 for small-to-mid lobar or capsulo-lenticular bleeds; lobar surgery matched this (13.4%) only in large clots. Thalamic evacuation was futile (82.3% mortality), and cerebellar decompression performed late still carried 54.5% mortality versus 16.6% medically. Multivariable analysis confirmed that low GCS, IVH, large hematoma volume, thrombocytopenia, and chronic alcohol use independently predicted in-hospital mortality. Limitations: This retrospective study lacked post-discharge functional outcome data (e.g., mRS at 90 days). Conclusions: This study presents the largest Romanian single-center ICH cohort, establishing national benchmarks and underscoring modifiable risk factors. Early ICH lethality aligns with Western data but is amplified by exposures such as alcohol misuse, anticoagulation, thrombocytopenia, and CKD. Priorities include preventive strategies, timely surgical access, wider adoption of minimally invasive techniques, and development of a prospective regional registry.

## 1. Introduction

Hemorrhagic stroke constitutes a minority of cerebrovascular events, yet contributes disproportionately to stroke-related mortality and long-term disability [1]. Among hemorrhagic strokes, spontaneous (non-traumatic) intracerebral hemorrhage (ICH)—bleeding directly into the brain parenchyma without underlying trauma—accounts for 10–15% of all strokes in Western populations and up to 25% in parts of Asia [2]. Case fatality remains approximately 35–40% at one month and 50–60% at one year despite advances in neurocritical care, imaging, and blood pressure management. Only one-quarter of survivors regain functional independence.

Prognosis after ICH depends on a constellation of factors. Five variables—age ≥ 80 years, hematoma volume ≥ 30 mL, admission GCS ≤ 12, infratentorial location, and intraventricular extension—form the validated ICH Score, powerfully discriminating mortality risk [3]. Additional predictors include pre-morbid anticoagulation, chronic kidney disease (CKD), hyperglycemia, thrombocytopenia, and heavy alcohol consumption [4]. Treatment options remain limited [5]. Intensive blood pressure reduction and rapid reversal of anticoagulation reduce hematoma expansion but have not translated into major outcome gains at population level. Randomized trials of open craniotomy (STICH I/II) failed to show an overall benefit except in anatomically superficial lobar clots, while minimally invasive techniques (MISTIE III) produced encouraging but inconclusive results [6,7,8]. Cerebellar evacuation is the only surgical indication strongly endorsed by guidelines [9].

Europe carries one of the world’s highest age-standardized stroke mortalities, yet national ICH registries are scarce. Romania’s age-adjusted stroke mortality in 2019 was 173 per 100,000—double the European Union mean [10]. Granular regional data are critical for tailoring stroke care, yet Romanian studies remain limited, typically consisting of small, brief cohorts. Radu et al. analyzed 53 patients over five months in Bucharest, reporting ~50% in-hospital mortality linked to hypertension, hematoma size, and deep hemorrhage [11]. Similarly, Ghelmez et al. studied 80 hypertensive ICH cases, finding a 39% 30-day mortality, associated with GCS, volume ≥ 30 cm^3^, IVH, and infratentorial location [12]. Our analysis significantly expands on these earlier efforts with a larger cohort (601 consecutive patients) over seven years, providing robust data for national benchmarks. The Sibiu County Clinical Emergency Hospital (SCCE H) is a 1200-bed tertiary referral center serving ~465,000 inhabitants of Sibiu County and neighboring rural districts. As the sole facility offering 24/7 neurosurgery, SCCE H receives all suspected ICH cases within a 75 km radius.

We present a comprehensive analysis of 601 consecutive adults with primary ICH admitted between January 2017 and December 2023. Our objectives were four-fold:

1. Describe demographic, clinical, and radiological characteristics using prospectively recorded variables without imputation or extrapolation.
2. Quantify in-hospital mortality overall and by predefined subgroups (age, sex, anatomical site, volume, treatment, comorbidities).
3. Compare outcomes with contemporary international literature, highlighting areas of concordance and divergence.
4. Identify modifiable factors relevant to regional stroke system planning and public health policy (e.g., anticoagulation practices, alcohol misuse).

By strictly adhering to the raw dataset supplied and avoiding assumptions, we aim to provide an authentic snapshot of ICH care in a middle-income European setting. While many prognostic factors we report align with established literature, we believe the regional variations in alcohol consumption, anticoagulant access, and delayed surgery make this cohort an important addition to global comparative epidemiology and may inform ICH care in similar healthcare systems internationally.

Although our center did not participate in major surgical trials, such as STICH II, which focused on the timing of open surgery, or MISTIE, which assessed minimally invasive evacuation via thrombolysis, these interventions are not standard practice worldwide Thus, their absence does not affect the core relevance of our study, which reflects real-world neurosurgical decision-making in a resource-constrained environment. Our findings help establish local benchmarks and inform region-specific treatment strategies.

## 2. Materials and Methods

### 2.1. Study Design and Ethical Approval

We conducted a single-center, retrospective, observational cohort study compliant with STROBE recommendations. The SCCE H institutional review board approved the protocol (reference SCCEH 2024 ICH 027), with waiver of individual consent owing to retrospective chart review, de-identification, and minimal risk.

### 2.2. Patient Selection

Using the electronic admission log and ICD 10 codes (I61.x), we identified all adults (≥18 years) diagnosed with ICH between 1 January 2017 and 31 December 2023.

Exclusion criteria were traumatic brain injury evident on history or imaging; vascular malformation (digital subtraction angiography or CTA/MRA) or angiographically proven aneurysm; cerebral tumor with hemorrhagic conversion; pure subarachnoid hemorrhage without intraparenchymal component; and patients declared dead on arrival before CT.

After exclusions, 601 consecutive cases constituted the analytic cohort.

### 2.3. Data Collection

Data were extracted from a prospectively maintained stroke database, including admission and discharge variables. Collected data encompassed demographics (age, sex, urban/rural status per National Institute of Statistics); medical history (hypertension, diabetes, CKD [eGFR < 60 mL/min/1.73 m^2^ or dialysis], chronic alcohol misuse [>3 units/day or AST/ALT > 2], prior antiplatelet or anticoagulant therapy, statin use, thrombocytopenia); clinical presentation (systolic BP, GCS, focal deficits, seizures); imaging (non-contrast CT within 30 min, hematoma volume [ABC/2], location [capsulo-lenticular, thalamic, lobar, cerebellar, brain stem, intraventricular], and IVH presence); treatment (conservative vs. surgery [craniotomy or posterior fossa decompression], timing from onset, indication); and outcomes (primary: in-hospital mortality; secondary: length of stay, discharge destination). Minimally invasive aspiration was not available.

Chronic alcohol misuse was defined by clinical documentation, daily intake ≥ 40 g (men) or ≥20 g (women), AST/ALT ratio > 2, or alcohol-related comorbidities. CKD was defined by prior diagnosis and/or eGFR < 60 mL/min/1.73 m^2^ on admission.

### 2.4. Data Quality Assurance

All numeric data underwent dual-entry verification by a dedicated three-member team consisting of a neurosurgeon, a resident, and a research fellow. Discrepancies (identified in approximately 2% of entries) were resolved by re-checking the original medical records or PACS imaging. Missing data were uncommon (<3%) for essential variables (age, sex, anatomical location, outcome), except for hematoma volume (26.5%), which could not be accurately measured for all patients due to inadequate or incomplete radiological documentation. Functional status at discharge was not consistently recorded.

### 2.5. Statistical Analysis

Descriptive statistics were used to summarize the cohort. Continuous variables are reported as mean ± SD or median (IQR), and categorical variables are reported as counts and percentages. Correlations (e.g., between volume and GCS) were explored using Spearman’s coefficient. Missing data were not imputed and are explicitly reported. Analyses were performed using SPSS v26.0 and Python (Statsmodels v0.14.0).

A multivariable logistic regression (complete case, *n* = 601) identified predictors of in-hospital mortality. Covariates included age, GCS, hematoma volume > 30 mL, IVH, CKD, anticoagulant or antiplatelet use, diabetes, thrombocytopenia, and chronic alcohol misuse. Odds ratios (ORs) with 95% confidence intervals (CIs) were reported. Statistical significance was set at *p* < 0.05.

Given the retrospective design, all associations are observational and non-causal.

## 3. Results

### 3.1. Baseline Demographics and Clinical Profile

The cohort comprised 601 consecutive adults with primary ICH. Men predominated (*n* = 359, 59.7%), presenting five years younger on average than women (66.0 vs. 71.9 y). Age skewed heavily toward older groups: almost four-fifths of patients were ≥60 years, and one in five was octogenarian (Table 1).

Geographically, the case mix reflected the county’s population distribution: 331 urban (55%) and 270 rural residents (45%). Rural patients were slightly younger (mean age 67.55 vs. 69.1 years for urban residents), with a similar gender distribution.

Cardiometabolic comorbidity was common. Documented hypertension affected 72% of the cohort, mirroring national health survey figures, and diabetes mellitus was present in 13.6% (nearly evenly divided by sex). Chronic kidney disease (CKD) and chronic alcohol misuse, each seen in roughly one in eight patients, were notable high-risk clusters; CKD was more frequent in women, whereas hazardous drinking was concentrated in men (17% of males vs. 5% of females).

Pre-morbid antithrombotic exposure was substantial: a total of 12.8% arrived on oral anticoagulants (roughly half warfarin, half DOACs), and 8.8% arrived on antiplatelet agents; a total of 6.5% had baseline thrombocytopenia. These profiles underscore the coexistence of hemorrhagic and thrombo-embolic risk in an ageing cerebrovascular population.

Initial stroke severity was high. The mean admission Glasgow Coma Scale (GCS) was 10.55, with men exhibiting deeper coma (10.24) than women (11.02). Mean hematoma volume was 30.4 mL, but one in four patients harbored hematomas > 30 mL, and the sex gap persisted (33.4 mL vs. 27.6 mL). Length of stay averaged 12 days (range 1–97 days), reflecting a mix of early fatalities and protracted neuro ICU courses.

### 3.2. Age Distribution, Severity, and Early Outcome

**Figure 1:**
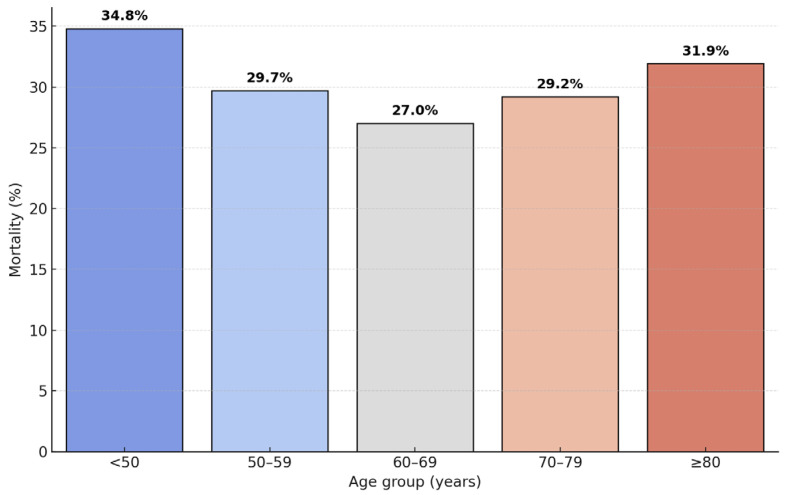
In-hospital mortality by age category (*N* = 601).

The cohort predominantly included older adults, with 464 patients (77.2%) aged ≥60 years and only 46 (7.6%) younger than 50, reflecting national demographic ageing trends. In-hospital mortality ranged non-linearly between 27.0% (ages 60–69 years) and 34.8% (<50 years, driven by large hematomas and delayed recognition), stabilizing near 30% among octogenarians (Table 2, Figure 1). Younger men presented with lower mean admission GCS due to large bleeds linked to hypertension and alcohol misuse, whereas older patients, especially women ≥ 75 years, more often had smaller lobar or amyloid-related hemorrhages influenced by anticoagulant use and age-related frailty. Male predominance peaked between ages 50 and 70 years, coinciding with higher alcohol use and uncontrolled hypertension. This pattern mirrors INTERACT pooled data, emphasizing the need for age-specific prevention: intensive hypertension and alcohol interventions for younger men and careful anticoagulant management for elderly patients [13].

### 3.3. Radiological Characteristics

**Figure 2:**
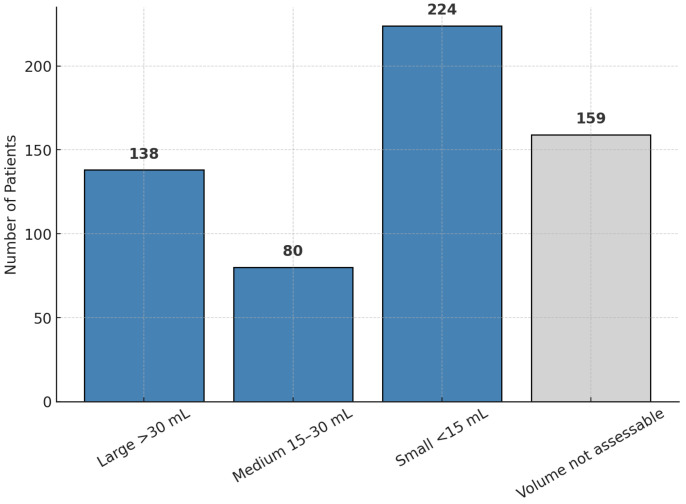
Hematoma volume categories (*n* = 601).

Volume spectrum: reliable CT-based volume calculations were available for 442 patients (73.5%). Nearly a quarter (23.0%) had large (>30 mL) hematomas, while 37.3% had small (<15 mL) bleeds (Table 3, Figure 2). Volume was strongly inversely correlated with admission GCS (Spearman ρ ≈ −0.62). The largest average volumes occurred in men younger than 50 years (mean 41 mL), contrasting notably with younger women (mean 25 mL).

**Figure 3:**
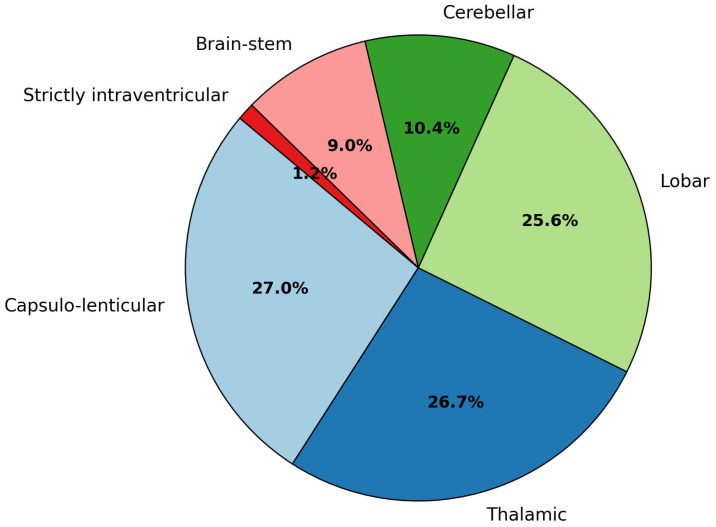
Anatomical distribution of ICH (*N* = 601).

Topographical pattern: deep nuclear locations (capsulo-lenticular and thalamic) each comprised roughly 25%, collectively representing half the cohort (50.6%). Lobar hemorrhages followed at 24.1%. Less frequent but critical were cerebellar (9.8%) and brain-stem (8.5%) hemorrhages. Strictly intraventricular hemorrhages were rare (1.2%) (Table 4, Figure 3).

Outcome by site: brain-stem hemorrhages had the highest mortality (74.5%), lowest GCS (mean 6.45), and largest mean volume (51 mL). Cerebellar hemorrhages had significantly lower mortality (23.7%). Lobar hemorrhages, despite larger volumes, had the lowest mortality (13.1%), reflecting their superficial location and higher initial GCS.

Intraventricular extension (IVH): IVH occurred in 241 patients (40.1%), who had larger mean volumes (45.2 vs. 22.1 mL), lower GCS (8.2 vs. 12.1), and significantly increased mortality (50.6% vs. 16.7%), emphasizing IVH’s prognostic importance.

### 3.4. Treatment Modality and Outcomes

Of 601 patients, 450 (74.9%) received medical management: mean hematoma 22.9 mL, GCS 11.0, mortality 125/450 (27.8%), LOS 12 days. Lobar (77 pts, 19.4 mL) and capsulo-lenticular (110 pts, 17.5 mL) bleeds showed the lowest fatality: 10/77 (13.0%) and 17/110 (15.4%), respectively. Thalamic ICH (135 pts, 20.5 mL) produced 39 deaths (28.9%); cerebellar (48 pts, 8.9 mL) produced eight deaths (16.6%); brain stem (51 pts, 51.4 mL, GCS 6.45) produced 38 deaths (74.5%).

Surgery was undertaken in 151 patients (25.1%): mean volume 57.5 mL, GCS 9.4, mortality 53/151 (35.1%), LOS 13.4 days. Lobar evacuation (67 pts, 48 mL, GCS 11.65) achieved nine deaths (13.4%). Capsulo-lenticular evacuation (45 pts, 68.9 mL) had 15 deaths (33.3%). Thalamic evacuation (17 pts, 63.8 mL, GCS 6.12) was least effective: 14 deaths (82.3%). Cerebellar decompression (11 pts, 21.1 mL, GCS 7.25) resulted in six deaths (54.5%).

**Figure 4:**
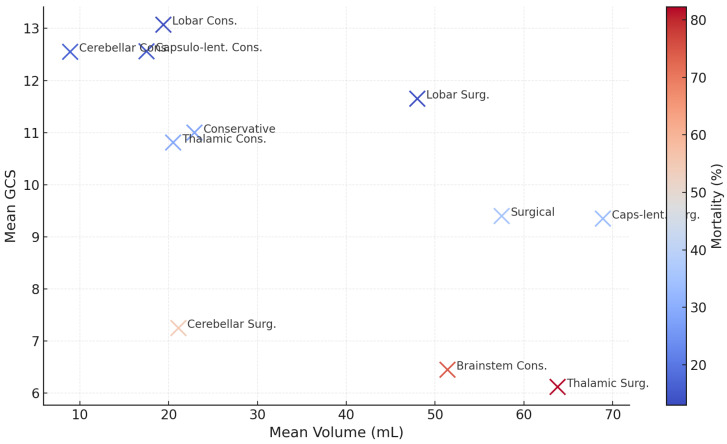
Mean hematoma volume vs. mean GCS (colored by mortality %).

**Figure 5:**
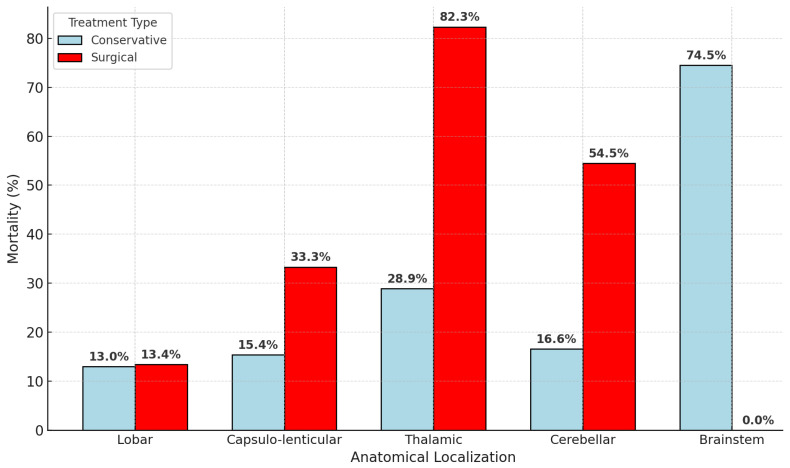
Mortality by anatomical localization and treatment modality.

These figures (Table 5, Figure 4 and Figure 5) indicate that medical therapy is adequate for most small-to-mid-volume lobar and capsulo-lenticular ICH; surgical benefit is clear in selected lobar cases but limited for deep thalamic hemorrhage, which retains >80% mortality despite intervention.

### 3.5. Risk Factor Subgroups

**Figure 6:**
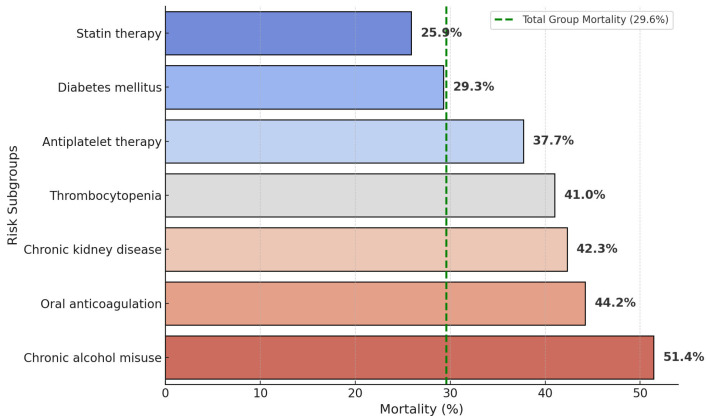
In-hospital mortality by risk subgroup.

Oral anticoagulants (12.8%, *n* = 77) were associated with high mortality (44.2%), reflecting larger hematoma volumes (mean 37 mL) and frequent intraventricular extension; DOAC vs. warfarin outcomes were not reliably distinguishable. Antiplatelet therapy (8.8%) also increased mortality (37.7%), underscoring its impact on hematoma expansion. Statin users (4.5%) had the lowest mortality (25.9%), aligning with observational data suggesting potential benefit or neutrality; survivor bias remains a limitation. Chronic alcohol misuse was present in 72 patients (12.0%), predominantly male, resulting in high mortality (51.4%) and younger mean age (62.7 y); notably, all patients combining alcohol misuse, warfarin, and thrombocytopenia died, highlighting severe combined risk. Diabetes mellitus (13.6%) had comparatively lower mortality (29.3%), suggesting hyperglycemia alone is less immediately harmful than coagulopathy or renal dysfunction; nevertheless, tight glycemic control remains crucial. Thrombocytopenia (<150 × 10^9^/L, 6.5%) correlated with high mortality (41.0%), consistent with known hematoma expansion risk; prophylactic transfusions were not routine due to clinical uncertainty. Chronic kidney disease (CKD, 11.8%) conferred substantial mortality (42.3%), attributable to uremic platelet dysfunction, hypertension, and electrolyte shifts, emphasizing the importance of early nephrology consultation and careful blood pressure control. Overall, systemic modifiable factors strongly influenced early prognosis beyond anatomical determinants (Table 6, Figure 6).

These unadjusted comparisons illustrate sizeable, statistically robust mortality gaps for anticoagulated, alcohol-using, and renally impaired patients—prioritizing rapid reversal, abstinence counselling, and nephrology input in acute ICH care [14].

### 3.6. Gender-Specific Findings

**Figure 7:**
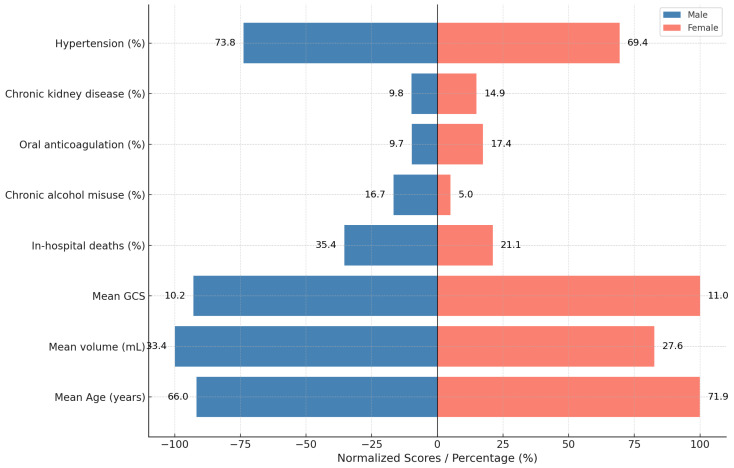
Gender-based clinical characteristics and outcomes.

Men (*n* = 359, mean age 66.0 years) constituted 60% of the cohort and differed from women (*n* = 242, mean age 71.9 years) on several key metrics (Table 7, Figure 7). They presented with larger hemorrhages (33.4 mL vs. 27.6 mL) and lower Glasgow Coma Scale (10.24 vs. 11.02), translating into a markedly higher in-hospital mortality of 35.4% versus 21.1%. The excess risk is statistically robust: risk ratio (RR) = 1.67 (95% CI 1.26–2.21, χ^2^ = 12.8, *p* = 0.0003). This paradox, higher male mortality despite younger age, highlights gender-specific differences in clinical severity and underlying risk profiles.

Anatomically, men were over-represented in brain-stem (9.2% vs. 7.4%) and capsulo-lenticular bleeds (28.7% vs. 20.7%), whereas women had a slightly higher share of thalamic ICH (28.1% vs. 23.1%). Risk-factor patterns were also divergent: chronic alcohol misuse was three-times more common in men (16.7% vs. 5.0%), while oral anticoagulant use predominated in women (17.4% vs. 9.7%). Thrombocytopenia and CKD were modestly more frequent among females.

Collectively, these data suggest that heavier alcohol exposure, poorer hypertension control, and earlier hypertensive arteriopathy drive larger, deeper hemorrhages in men, whereas in older women, cerebral amyloid angiopathy and anticoagulant use become dominant contributors.

### 3.7. Residence and Pre-Hospital Delay

Urban patients (*n* = 331) displayed larger mean hematoma volumes (33.3 mL vs. 28.34 mL), slightly lower admission GCS (10.38 vs. 10.78), and higher in-hospital mortality (32.9% vs. 25.6%) compared to rural counterparts (*n* = 270). Urban patients were also older on average (69.1 years vs. 67.55 years). Additionally, urban residents had greater use of oral anticoagulants (40 urban vs. 37 rural), antiplatelet therapy (27 urban vs. 26 rural), and statins (18 urban vs. 9 rural). Chronic kidney disease was more frequent in urban patients (44 urban vs. 27 rural), whereas chronic alcohol misuse showed similar distribution (40 urban vs. 32 rural) (Table 8).

These findings indicate two converging mechanisms: pre-hospital attrition of the most catastrophic rural bleeds lowers observed rural severity, whereas a heavier chronic-disease and antithrombotic burden in cities yields larger, deadlier urban hemorrhages despite faster arrival. The distinct demographic and risk-factor profiles, therefore, call for urban-focused interventions—tight outpatient blood-pressure control, vigilant anticoagulant and CKD management, and aggressive cardiovascular risk modification—to offset the excess mortality seen in metropolitan patients.

### 3.8. Multivariable Logistic Regression Analysis

We performed a multivariable logistic regression using complete-case data (*n* = 601) to assess independent predictors of in-hospital mortality in patients with spontaneous ICH. The model included ten clinically relevant variables: age, Glasgow Coma Scale (GCS) score, hematoma volume > 30 mL, intraventricular hemorrhage (IVH), chronic kidney disease (CKD), oral anticoagulation (OAC), antiplatelet therapy, diabetes mellitus, thrombocytopenia, and chronic alcohol use.

The final model identified the following independent predictors: lower GCS (OR 0.67, 95% CI 0.62–0.73, *p* < 0.001), IVH (OR 2.87, 95% CI 1.88–4.38, *p* < 0.001), hematoma volume > 30 mL (OR 1.80, 95% CI 1.11–2.90, *p* = 0.016), thrombocytopenia (OR 1.75, 95% CI 1.10–2.85, *p* = 0.021), and chronic alcohol use (OR 1.59, 95% CI 1.00–2.52, *p* = 0.049).

Other variables including age, CKD, anticoagulant or antiplatelet use, and diabetes were not statistically significant after adjustment. These results emphasize the prognostic value of neurological status and imaging findings over systemic comorbidities in predicting early mortality in ICH.

## 4. Discussion

Early mortality in this 601-patient cohort was 29.6%, matching the 26–33% reported in recent EU registries and slightly below the ≈35% global average [15]. Outcomes were driven primary by the classic quartet: brain-stem site, volume > 30 mL, intraventricular extension, and low GCS—closely replicating multicenter observations [16].

While our findings reaffirm established prognostic factors—hematoma volume, infratentorial location, and low GCS—they also highlight distinct differences in demographics, risk exposures, and care access compared to Western or East Asian cohorts. Notably, higher rates of chronic alcohol misuse among middle-aged men and delays in cerebellar surgery reflect context-specific challenges. These variations underscore the importance of regionally informed strategies in global stroke care.

Men accounted for approximately 60% of admissions and, despite a mean age five years younger than women (66.0 vs. 71.9 years), presented with larger hematomas, deeper coma, and significantly higher mortality (35.4% vs. 21.1%; RR = 1.67) [17]. This excess male risk surpasses most pooled estimates and is likely driven by modifiable exposures, including heavier alcohol consumption, poorer hypertension control, and earlier onset hypertensive arteriopathy, consistent with findings from sex-stratified reviews.

Minimally invasive surgical (MIS) techniques for ICH evacuation—such as endoscopic aspiration or stereotactic thrombolysis—remain rarely used in most neurosurgical departments worldwide. Although not significantly more costly than craniotomy, MIS requires dedicated equipment and neurosurgical training. Importantly, randomized trials have shown only modest or inconsistent benefits, with outcomes varying by location and timing. Given these uncertainties, large-scale adoption is premature. However, selective implementation in high-volume centers may be justified through structured pilot programs.

Male predominance peaked between ages 50 and 70 years, coinciding with the highest alcohol misuse and uncontrolled hypertension. Female representation sharply increased after 75 years, reflecting higher anticoagulant use and cerebral amyloid angiopathy prevalence. Early mortality remained lower in women, likely due to smaller hematoma volumes.

Brain-stem hemorrhage remained the deadliest subtype (74.5%), echoing contemporary Chinese series and highlighting the absence of effective interventions [18].

Alcohol-related ICH accounted for 12% of cases but exceeded 50% mortality, well above Western figures; Romania’s high per capita alcohol consumption identifies an urgent preventive target [19]. Anticoagulant-associated ICH retained a 44% lethality despite DOAC uptake, paralleling Japanese data (DOAC ≈ 40%, warfarin ≈ 55%); intermittent access to idarucizumab and PCC exposes a supply chain gap [20].

Comorbid chronic kidney disease (CKD) carried a mortality of 42%, aligning closely with pooled international analyses (adjusted OR 1.74) highlighting CKD as an independent predictor of a poor outcome [21,22,23]. Thrombocytopenia similarly conferred substantial lethality (41%), consistent with North American registries, although the role of prophylactic platelet transfusion remains unclear. Notably, chronic alcohol misuse was documented in 12% of patients, three-fold more frequent among men, and associated with a 51.4% mortality, mirroring the excess death risk observed in multi-ethnic cohorts such as ERICH [24,25]. The intersection of alcohol abuse and anticoagulant therapy was uniformly fatal in all four cases exhibiting these combined risk factors, underscoring the critical need for targeted preventive strategies, including alcohol cessation, careful anticoagulant management, and early platelet optimization [26,27].

Rural patients showed smaller hematoma volumes and better GCS but similar mortality, likely reflecting pre-hospital death of ultra-severe cases and delayed transfers. The slightly lower rural mortality may reflect selection bias, as longer transport times limit hospital admission to more stable patients. Urban cases, by contrast, include a broader severity spectrum due to faster access. These findings highlight the need for better pre-hospital triage and transfer protocols to address regional disparities in ICH outcomes [28].

Large hematoma volume > 30 mL, depressed Glasgow Coma Scale, infratentorial location, and intraventricular extension co-segregated with the highest early lethality in our cohort, faithfully reproducing the ICH-Score constellation first described by Hemphill and colleagues. Brain-stem bleeds were almost uniformly fatal (38/51, 74.5%), identical to pooled meta-analytic estimates of 70–80%.

Intracerebral hemorrhage with ventricular spread occurred in 40% of cases and doubled crude mortality (50.6% vs. 16.7%), mirroring the independent risk attributed to IVH in large registry analyses [29].

Conservative treatment sufficed for most lobar and small-to-mid-volume deep bleeds (overall 27.8% mortality), whereas surgical benefit was confined to carefully selected lobar evacuations (13.4% mortality), echoing the neutral results of STICH-II [7].

Open surgery for deep thalamic hemorrhage showed poor outcomes (82.3% mortality), consistent with a limited benefit in this location. Cerebellar decompression, though guideline-recommended for mass effect or brain-stem compression, still carried a 54.5% mortality in our cohort. Delays beyond the 24–48 h therapeutic window were common and likely contributed to this. These findings underscore the need for earlier detection, prompt neurosurgical evaluation, and faster transfer protocols for posterior fossa ICH.

Our multivariable analysis confirmed that low GCS, intraventricular hemorrhage, and large hematoma volume are strong independent predictors of in-hospital mortality, aligning with prior large-scale ICH studies. Thrombocytopenia and chronic alcohol use also remained significant after adjustment, underscoring the role of hematologic vulnerability. Conversely, age, diabetes, CKD, and antithrombotic therapy—despite showing associations in univariate analysis—did not retain significance, likely due to collinearity with radiologic or neurologic severity. These findings reaffirm the dominant prognostic value of early clinical status and hematoma burden.

### 4.1. Study Strengths

This study is based on a large, consecutive, county-wide cohort, with prospectively coded variables, dual data verification, and no imputation—yielding a robust and reliable Romanian dataset. As the sole neurosurgical provider in the region, SCCEH ensures that the sample reflects the full local ICH burden, enhancing its representativeness and value for future comparisons. Inclusion of both surgically and conservatively treated patients across all ICH locations (lobar, deep, infratentorial, IVH) allows for comprehensive stratified analyses and real-world insights into treatment patterns and outcomes in a mixed tertiary cohort.

### 4.2. Limitations

The retrospective design, lack of post-discharge functional outcomes (e.g., mRS at 30 or 90 days), and single-center scope limit generalizability and long-term inference. The absence of functional follow-up stems from inconsistent tracking systems and logistical constraints. Some multivariable models could not be performed for all outcomes. Improving outcomes in this setting will require faster rural triage, structured alcohol cessation programs, 24/7 anticoagulation reversal access, adoption of minimally invasive evacuation techniques with multicenter training, and early screening for CKD and thrombocytopenia.

## 5. Conclusions

This seven-year study of 601 consecutive ICH cases—the largest Romanian cohort to date—found an in-hospital mortality of 29.6%, driven by classic predictors: GCS ≤ 10, volume > 30 mL, IVH, and infratentorial location (brain-stem mortality 74.5%). Men had higher mortality than women (35.4% vs. 21.1%) despite being younger, likely due to deeper bleeds and alcohol misuse. Systemic risk factors, such as alcohol use (51%), anticoagulation (44%), thrombocytopenia (41%), and CKD (42%), amplified lethality. Conservative treatment sufficed for most supratentorial bleeds; lobar surgery offered similar outcomes only in large clots. Thalamic evacuation was ineffective, and delayed cerebellar decompression showed high mortality (54.5%), likely reflecting case severity. These findings underscore the need for faster triage, tailored surgical selection, and prospective studies with functional follow-up to improve outcomes.

These findings establish a national benchmark and highlight key priorities: community-level blood pressure and alcohol reduction programs (particularly for middle-aged men), around-the-clock access to reversal agents and platelet optimization, early nephrology input for CKD, refined surgical selection and timing for deep and posterior fossa ICH, and gradual implementation of minimally invasive techniques. A prospective multicenter registry with standardized 90-day outcomes—including mRS, rebleeding, rehospitalization, and mortality—will be crucial to assess intervention impact and inform national ICH care pathways. While locally rooted, these results offer a valuable point of comparison for other Central and Eastern European or middle-income health systems.

## Figures and Tables

**Table 1:** Baseline characteristics of 601 patients with spontaneous ICH (2017–2023).

Characteristic	All (*n* = 601)	Male (*n* = 359)	Female (*n* = 242)
Age, years—mean (SD)	68.4	66.0	71.9
<60 y, *n* (%)	137 (22.8)	98 (27.3)	39 (16.1)
≥80 y, *n* (%)	119 (19.8)	49 (13.6)	70 (28.9)
Residence—*n* (%)			
Urban	331 (55.1)	197 (54.9)	134 (55.4)
Rural	270 (44.9)	162 (45.1)	108 (44.6)
Key comorbidities—*n* (%)			
Hypertension	433 (72.0)	265 (73.8)	168 (69.4)
Diabetes mellitus	82 (13.6)	44 (12.3)	38 (15.7)
Chronic kidney disease	71 (11.8)	35 (9.8)	36 (14.9)
Chronic alcohol misuse	72 (12.0)	60 (16.7)	12 (5.0)
On oral anticoagulants	77 (12.8)	35 (9.7)	42 (17.4)
On antiplatelet agents	53 (8.8)	34 (9.5)	19 (7.9)
Thrombocytopenia (<150 × 10^9^/L)	39 (6.5)	20 (5.6)	19 (7.9)
Stroke severity			
Admission GCS—mean	10.55	10.24	11.02
Hematoma volume, mL—mean	30.4	33.4	27.6
Volume > 30 mL, *n* (%)	138 (23.0)	83 (23.1)	55 (22.7)
Hospital stay, days—mean (range)	12 (1–97)	12 (1–57)	12 (1–97)

**Table 2:** Age-stratified patient numbers, deaths, and in-hospital mortality.

Age Group (Years)	Patients, *n*	Deaths, *n*	Mortality %
<50	46	16	34.8
50–59	91	27	29.7
60–69	174	47	27.0
70–79	171	50	29.2
≥80	119	38	31.9
Total	601	178	29.6

**Table 3:** Hematoma volume categories.

Volume Category	Patients (*n*)	Percentage of Cohort (%)
Large > 30 mL	138	23.0
Medium 15–30 mL	80	13.3
Small < 15 mL	224	37.3
Volume not assessable	159	26.5

**Table 4:** Anatomical distribution and outcomes.

Site	Patients (*n*)	Mean Volume (mL) ± SD	Mean GCS
Capsulo-lenticular	153	33.0 ± 41.4	11.3 ± 4.0
Thalamic	151	25.53 ± 39.5	10.12 ± 4.4
Lobar	145	33.8 ± 29.6	12.44 ± 3.0
Cerebellar	59	10.85 ± 12.2	11.45 ± 4.8
Brain stem	51	51.4 ± 74.5	6.45 ± 4.6
Strictly intraventricular	7		12.5 ± 4.3

**Table 5:** Treatment modality and in-hospital mortality.

Treatment Group	Patients (*n*)	Mean Volume (mL)	Mean GCS	Deaths (*n*)	Mortality %	Mean LOS (Days)
Conservative	450	22.9	11.0	125	27.8%	12.0
Lobar	77	19.4	13.07	10	13.0%	11.06
Capsulo-lenticular	110	17.5	12.56	17	15.4%	13.6
Thalamic	135	20.5	10.81	39	28.9%	12.80
Cerebellar	48	8.9	12.55	8	16.6%	11.36
Brain stem	51	51.4	6.45	38	74.5%	9.38
All surgical	151	57.5	9.4	53	35.1%	13.4
Lobar evacuation	67	48.0	11.65	9	13.4%	14.8
Capsulo-lenticular evacuation	45	68.9	9.35	15	33.3%	12.76
Thalamic evacuation	17	63.8	6.12	14	82.3%	10.89
Cerebellar evacuation	11	21.1	7.25	6	54.5%	12.73

**Table 6:** Risk-factor subgroups and mortality.

Risk Subgroup	Patients (*n*)	Deaths (*n*)	Mortality (%)	Mortality Without Risk (%)	Risk Ratio (RR)	*p* (χ^2^)
Oral anticoagulation	77	34	44.2	27.5	1.61	0.003
Antiplatelet therapy	53	20	37.7	28.8	1.31	0.16
Statin therapy	27	7	25.9	29.8	0.87	0.69
Chronic alcohol misuse	72	37	51.4	26.7	1.93	<0.001
Diabetes mellitus	82	24	29.3	29.7	0.99	0.98
Chronic kidney disease	71	30	42.3	27.9	1.42	0.11
Thrombocytopenia (<150 × 10^9^ L^−1^)	39	16	41.0	28.8	1.42	0.013

Interpretation: Oral anticoagulation, chronic alcohol misuse, and CKD all double or near-double the odds of death (RR 1.5–1.9, *p* ≤ 0.013). Thrombocytopenia shows a non-significant 42% relative increase (RR 1.42, *p* = 0.11). Antiplatelet use and diabetes do not significantly shift mortality, while statin exposure trends toward neutral or slightly lower risk.

**Table 7:** Sex-specific characteristics and outcomes.

Variable	Male (*n* = 359)	Female (*n* = 242)
Average age (years)	66.0	71.9
Mean volume (mL)	33.4	27.6
Mean GCS	10.24	11.02
In-hospital deaths *n* (%)	127 (35.4%)	51 (21.1%)
Chronic alcohol misuse *n* (%)	60 (16.7%)	12 (5.0%)
Oral anticoagulation *n* (%)	35 (9.7%)	42 (17.4%)
Chronic kidney disease *n* (%)	35 (9.8%)	36 (14.9%)
Blood hypertension *n* (%)	265 (73.8%)	168 (69.4%)

RR for death (male vs. female) = 1.67, *p* = 0.0003.

**Table 8:** Residence, pre-hospital delay, and outcome.

Variable	Urban	Rural
Patients (*n*)	331	270
Mean volume (mL)	33.3	28.34
Mean age, year	69.1	67.6
Mean GCS	10.38	10.78
In-hospital deaths (*n*)	109	69
Mortality (%)	32.9	25.6

## Data Availability

DOI: 10.5281/zenodo.15368709.

## Abbreviations

The following abbreviations are used in this manuscript:

ATACH	Antihypertensive Treatment in Acute Cerebral Haemorrhage trial
BP	Blood pressure
CI	Confidence interval
CKD	Chronic kidney disease
CT	Computed tomography
CTA/MRA	CT angiography/Magnetic-resonance angiography
DOAC	Direct oral anticoagulant
GCS	Glasgow Coma Scale
ICH	Intracerebral hemorrhage
ICU	Intensive-care unit
INTERACT	Intensive Blood Pressure Reduction in Acute Cerebral Haemorrhage trial
IVH	Intraventricular hemorrhage
LOS	Length of hospital stay
mRS	Modified Rankin Scale
MISTIE	Minimally Invasive Surgery plus rt-PA for ICH Evacuation trial
PCC	Prothrombin complex concentrate
RR	Risk ratio
SAH	Subarachnoid hemorrhage
SBP	Systolic blood pressure
SCCE H	Sibiu County Clinical Emergency Hospital
STICH	Surgical Trial in IntraCerebral Haemorrhage (I/II)
STROBE	Strengthening the Reporting of Observational Studies in Epidemiology
